# Thyroid transcription factor-1 positive primary breast cancer: a case report with review of the literature

**DOI:** 10.1186/1746-1596-5-37

**Published:** 2010-06-17

**Authors:** Tor A Klingen, Ying Chen, Marian D Gundersen, Hans Aas, Bjørn Westre, Torill Sauer

**Affiliations:** 1 Department of Pathology, Vestfold County Hospital, Halfdan Wilhelmsens Alle' 17, N-3116 Tønsberg, Norway; 2 Department of Radiology, Vestfold County Hospital, Halfdan Wilhelmsens Alle' 17, N-3116 Tønsberg, Norway; 3 Department of Surgery, Vestfold County Hospital, Halfdan Wilhelmsens Alle' 17, N-3116 Tønsberg, Norway; 4 Department of Pathology, Ålesund Hospital, Åsehaugen 5, N-6017 Ålesund, Norway; 5 Department of Pathology, Ullevål University Hospital, Kirkeveien 166, N- 0407 Oslo, Norway

## Abstract

This case describes an infiltrating breast tumour with thyroid transcription factor-1 (TTF-1) positive staining and ductal differentiation in a 72-year-old woman. The presence of ductal carcinoma in situ with positive TTF-1 is a strong indication that this is a primary tumour and not a metastasis from lung.

On PET scan and CT follow up there were no other tumours found in this patient. We are not aware of any previously reported TTF-1 positive primary breast carcinoma with ductal differentiation.

## Background

TTF-1 is a tissue-specific transcription factor expressed in epithelial cells of the lung and the thyroid, including C-cells as well as certain areas of the brain. TTF-1 is expressed in approximately 72% of pulmonary adenocarcinomas[1], and may be a valuable marker for identifying the lung as the site of origin of a metastatic adenocarcinoma.

## Case Presentation

A 72-year-old gravida 1/para 1 woman presented with a palpable mass in the left breast.

Her sister died of breast cancer some years earlier. There was a past history of operated parathyroid adenoma some years ago but no previous history of any malignancy. The patient was a non- smoker and had never used hormonal drugs.

Clinical examination demonstrated a palpable tumour in her left breast in the upper inner quadrant, no fixation to skin or underlying fascia.

Mammography from upper inner quadrant of the left breast showed a dense, relatively well defined, oval shaped mass lesion with a microlobulated margin measuring 51 × 33 × 35 mm with coarse calcifications centrally in the mass and in addition finer micro calcifications antero lateral to the main tumour, malignant in appearance. The total maximal diameter including the calcifications was approximately 6 cm.

Ultrasound left breast showed an irregular solid malignant appearing tumour, corresponding to the mammographic lesion, measuring approximately 38 mm maximal diameter. No enlarged lymph nodes in the left axillae. PET scan and CT scan showed no other malignancy other than known breast tumour.

As no other origin for the tumour was found, the patient was treated with left mastectomy and sentinel lymph node mapping. The sentinel node was normal. The patient had an uncomplicated recovery and was discharged the next day following the operation.

## Materials and methods

We performed immunohistochemical investigation using the indirect streptavidin-biotin method on 3-5 μm slices. Regarding TTF-1 staining we used same methods at two different laboratories for verification of the findings. Further staining for CK5/6, CK 7, CK20, CK 18, mammaglobin, GCDFP-15, ER, PGR, chromgranin, synaptophysin, CD 56, HER 2/neu, Ki67, p 63 and MA was performed (table 1).

**Table 1:** Immunohistochemistry

Antibody	Clone	Dillution	Company
TTF-I	SPT24	1:100	Novocastra

CK5/6	D5/16B4	1:800	Chemicon

CK7	OV-TL-12/30	1:100	DAKO

CK20	Ks20.8	Ready to use	Roche

CK18	DC10	1:40	DAKO

Mammaglobin	304-1A5	1:150	DAKO

GCDFP-15	23A3	1:200	Novocastra

ER	SP1	Ready to use	Roche

PGR	1E2	Ready to use	Roche

Chromogranin	LK2H10	Ready to use	Roche

Synaptophysin	27G12	1:80	Novocastra

CD56	1B6	1:50	Novocastra

Her2/neu	4B5	Ready to use	Roche

Ki67	30.9	Ready to use	Roche

P63	4A4	Ready to use	Roche

MA	HUC1-1	Ready to use	Roche

## Results

**Figure 1:**
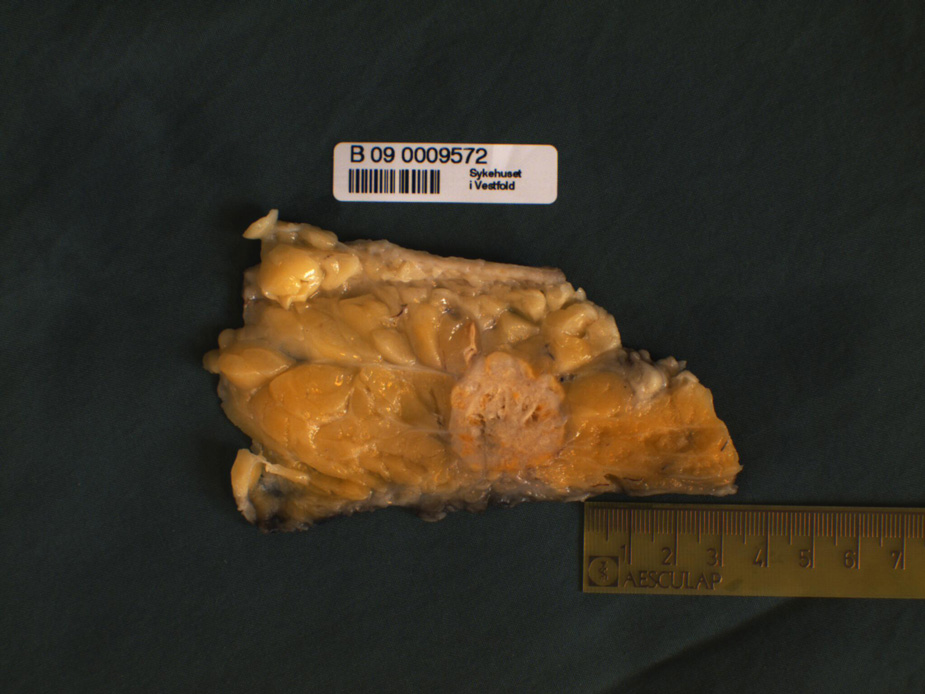
**Gross specimen showing well-circumscribed deep seated tumour with yellow necrotic areas**.

Macroscopic investigation showed a mastectomy specimen measuring 21 × 18 × 3 cm, weighing 1042 gram. In the upper inner quadrant there was a round, well-circumscribed tumour with largest diameter 3,8 cm laying 0,6 cm from the chest wall. The tumour was white with pale yellow necrotic areas (Figure 1).

**Figure 2:**
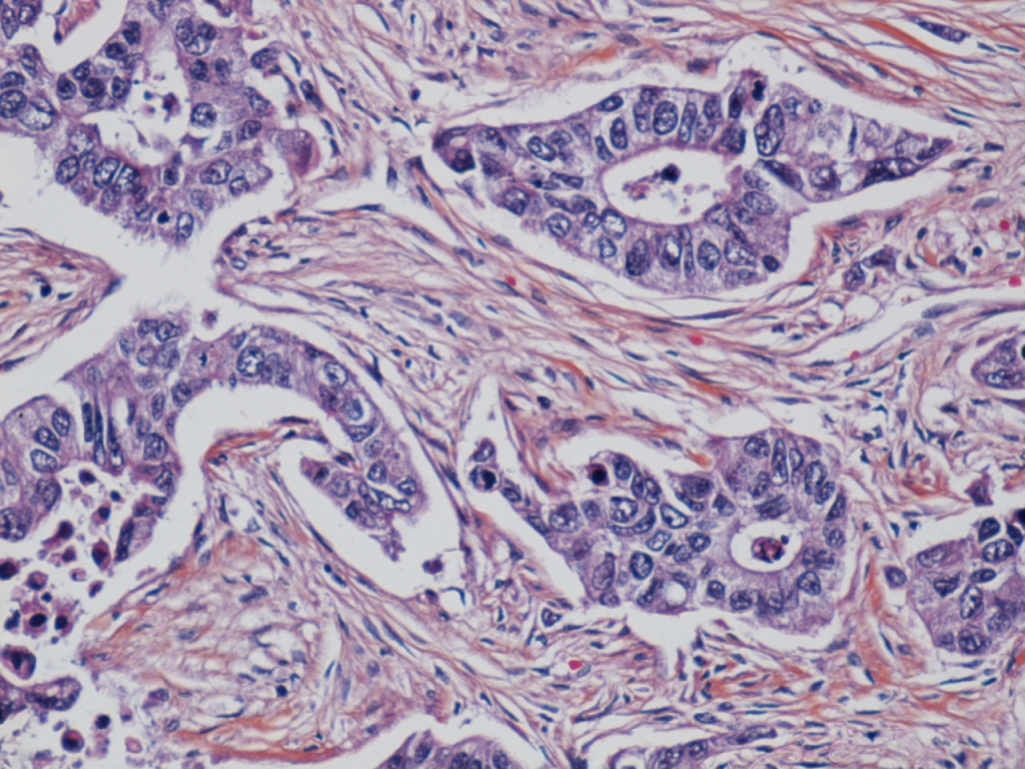
**Tumour with glandular elements, vesicular nuclei and mitoses**.

Microscopic investigation showed a sharp demarcation of the tumour to the surrounding breast tissue. There was some necrosis centrally, with more preserved structure peripherally in the tumour. The tumour tissue grew as trabeculae and confluent nests with scanty glandular tissue. The tumour cells were large with moderate amount of cytoplasm. The nuclei were variable in appearance being round, oval or spindle shaped. The chromatin was coarsely granulated and vesicular. There were numerous mitoses and apoptotic bodies (Figure 2).

**Figure 3:**
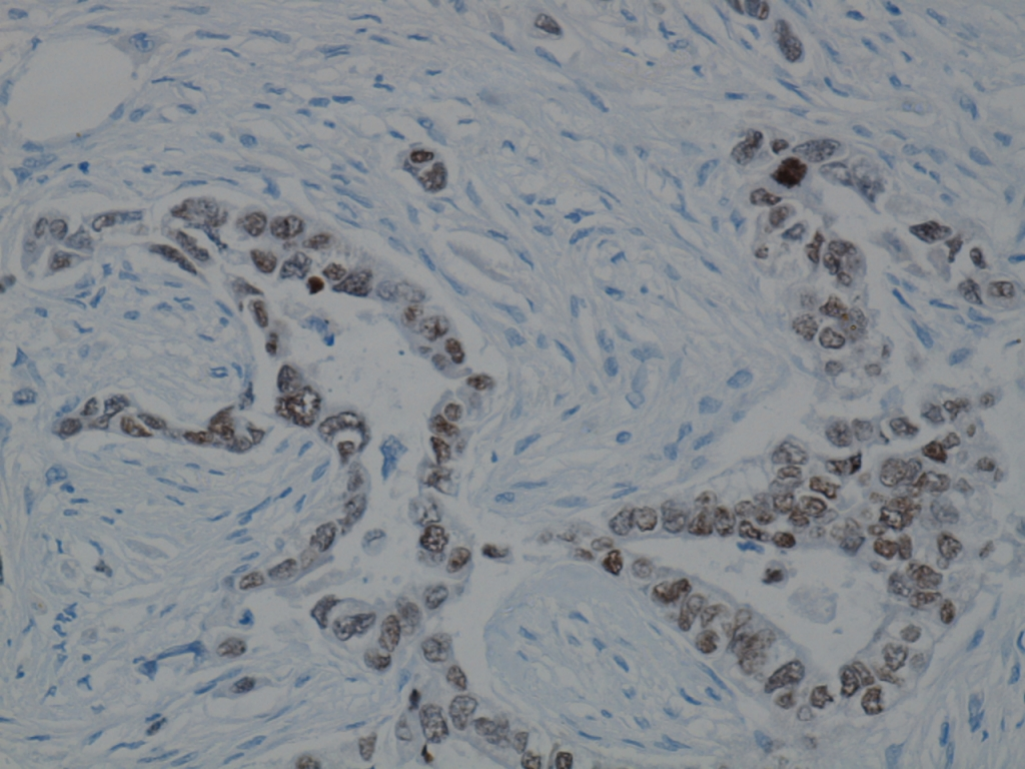
**Invasive glandular tumour area is immunoreactive for TTF-1**.

**Figure 4:**
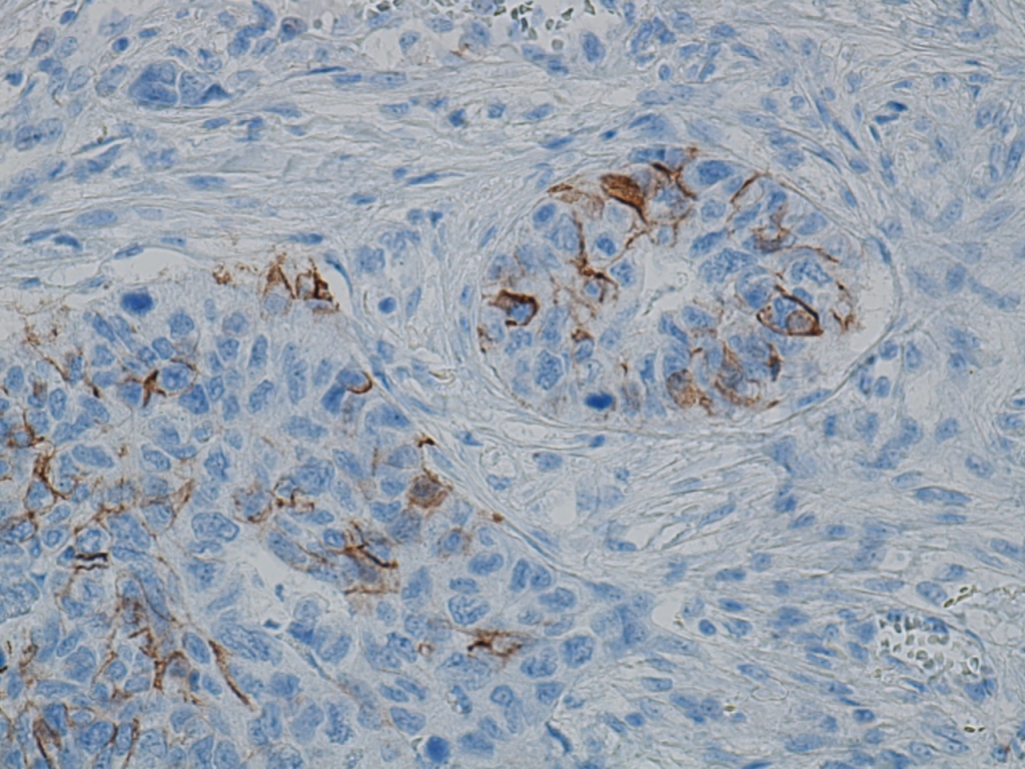
**CD56 immunoreactivity in a focal invasive tumour area**.

**Figure 5:**
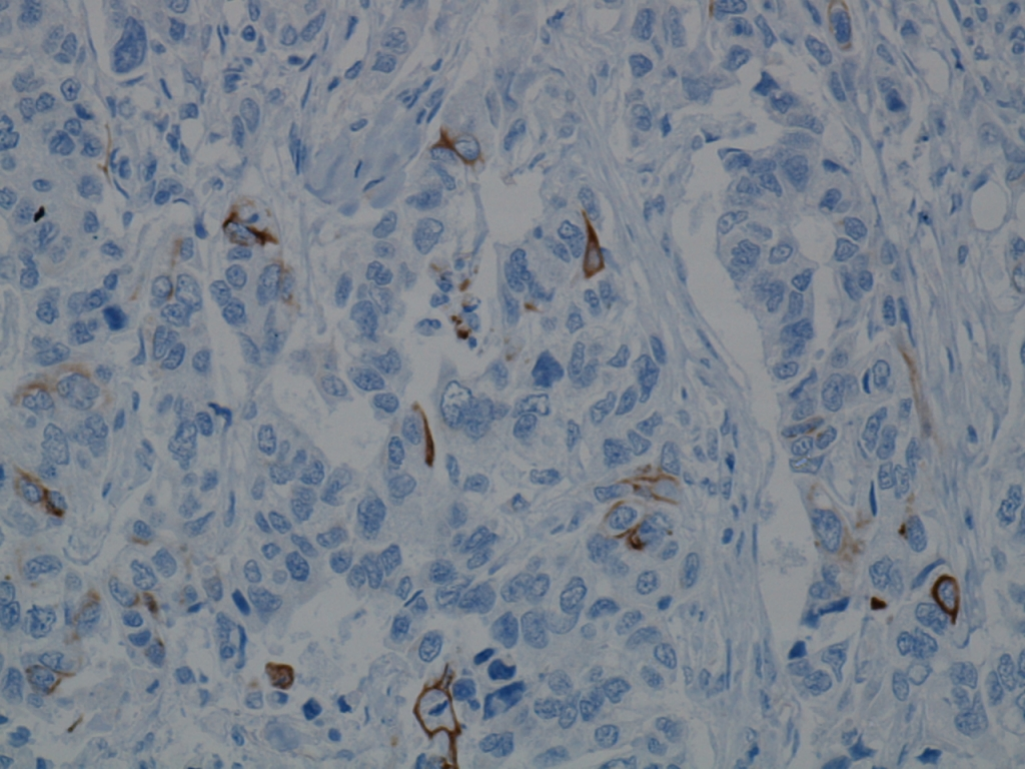
**CK5/6 immunoreactivity demonstrated in isolated cells within the invasive tumour**.

The nuclei in the invasive tumour cells showed diffuse and strong positive immunoreactivity to TTF-1 marker (Figure 3). The neuroendocrine marker CD56 was slightly positive (Figure 4), whilst chromogranin and synaptophysin were negative. CK 5/6 was demonstrated in isolated cells within the tumour (Figure 5). Ki67 was positive in over 50% of the tumour cells. The tumour was positive for CK7 and CK18 but negative for ER, mammaglobin, GCDPF-15 and CK20. HER2/neu showed heterogeneous C-erb-2 protein score 2+/3+, but silver in situ hybridization (SISH) without gene amplification. There was a weak positive reaction to PGR in the biopsy material, but not in the mastectomy tissue.

**Figure 6:**
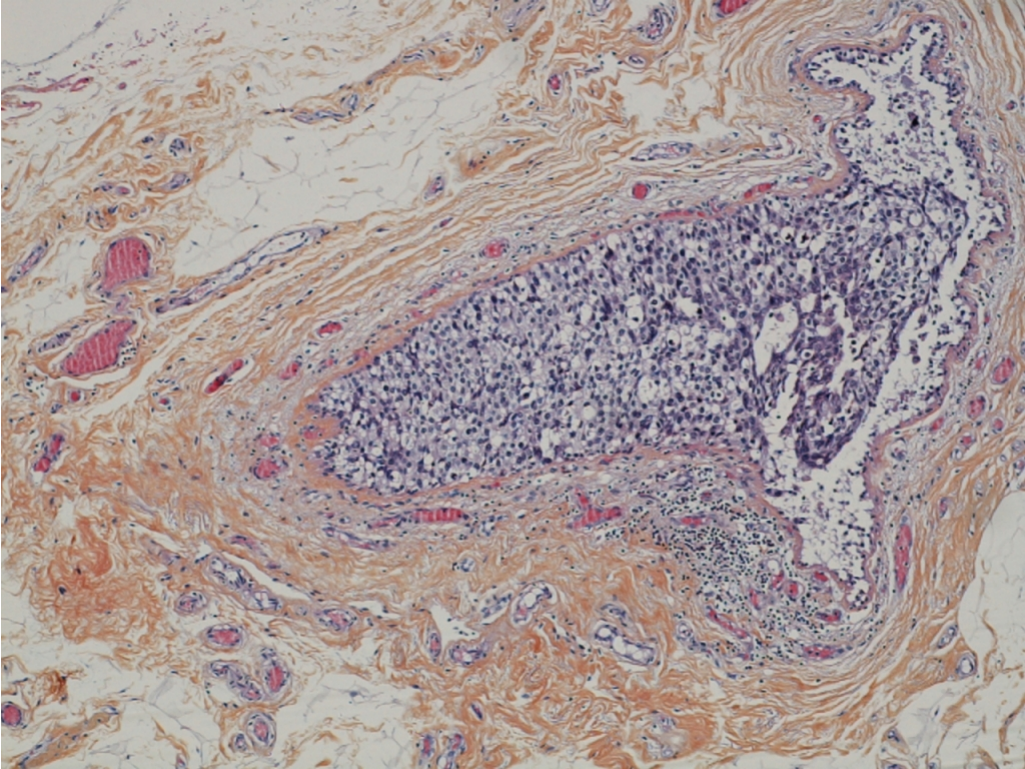
**Ductal carcinoma in situ in vicinity of the tumour**.

**Figure 7:**
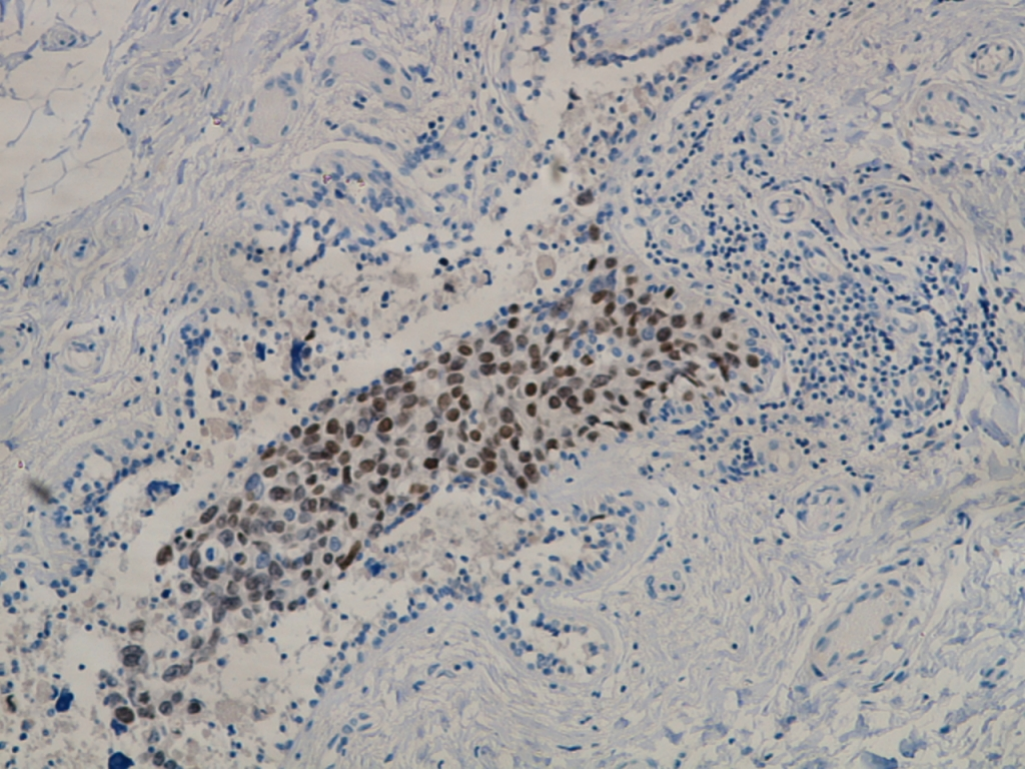
**Ductal carcinoma in situ with TTF-1 immunoreactivity**.

**Figure 8:**
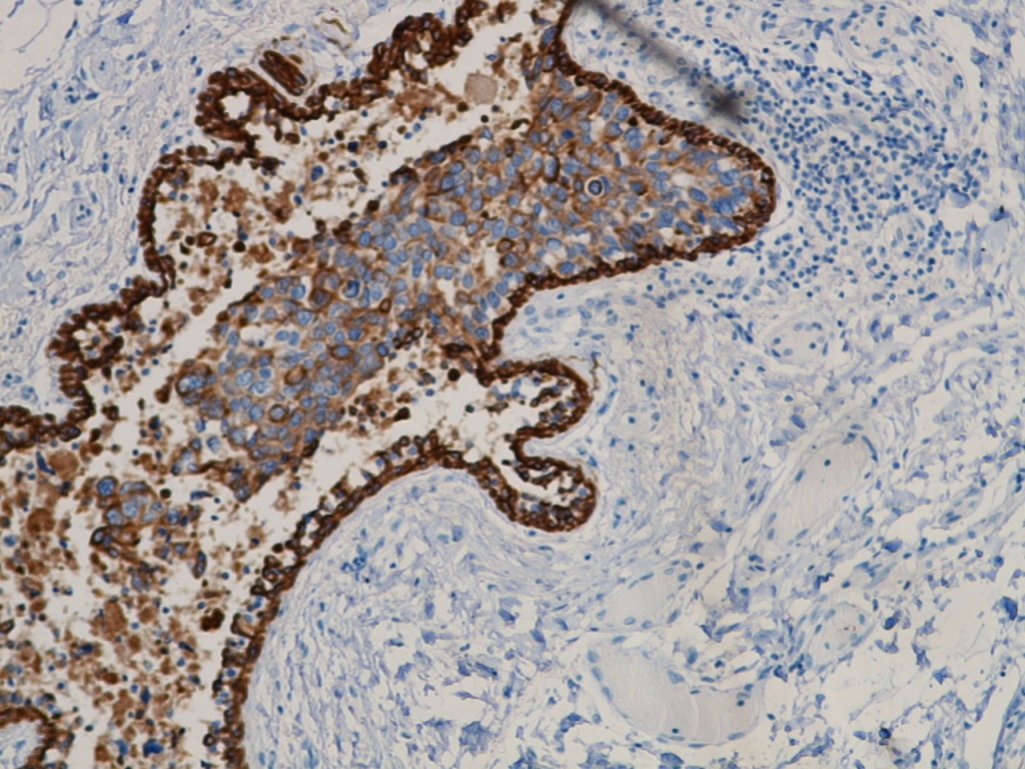
**Ductal carcinoma in situ with CK 5/6 immunoreactivity**.

An in situ component was seen in the vicinity of the tumour. The myoepithelial layer of the carcinoma in situ was verified immunohistochemically with p63 and MA. The in situ tumour cells had the same morphological appearance as in the invasive tumour (Figure 6). The in situ component showed a positive reaction to TTF-1(Figure 7) and CK5/6 (Figure 8).

## Discussion

We believe this case represents TTF-1 positive infiltrating ductal breast carcinoma, histological grade 3. The tumour was heterogeneous with small areas of basal-like and neuroendocrine differentiation.

In three large studies of primary breast carcinomas [1-3] there were 96, 51 and 35 (total 182) carcinoma cases, respectively, that were all TTF-1 negative. Only a few cases of TTF-1 primary small-cell carcinoma in breast have been reported [4,5]. Yamamoto described two small-cell breast carcinomas with positive TTF-1 whilst Ersahin reported one case of small-cell breast carcinoma which was positive for TTF-1 and basal-like markers. In the case we report, we found no areas typical for small cell carcinoma growth, although some areas with trabecular growth gave a partial neuroendocrine impression. Because of heterogeneous growth with focally oval and spindled shaped nuclei, a metaplastic carcinoma was considered. It is difficult to rule out this alternative, but a lack of squamous or heterologous metaplasia makes this less possible.

The initial investigations of the biopsy gave suspicion of metastasis from lung cancer because of the positive TTF-1 reaction. Previous studies have shown that lung cancer, malignant melanoma and lymphoma are the three most common candidates for secondary breast tumours [6-8]. An almost round tumour could resemble a metastasis, but some high grade primary tumours can also have this shape. In our case the tumour was deep seated in the breast (6 mm from the chest wall). This correlates poorly with secondary breast tumours which are often found near to the skin [6,8].

The presence of a positive TTF-1 reaction in the in situ component as well as in the invasive component in this case gives strong support for primary breast carcinoma. We are not aware of any previously reported TTF-1 positive ductal carcinoma in situ of non small-cell type. In order to be quite sure that this represented breast ducts and not vessels with tumour tissue or peritumoral retraction artefacts, we also investigated these areas with MA and p63. Both markers were positive for myoepithelial cells.

Mammaglobin and GCDFP-15 were negative in the tumour, but this does not exclude primary breast carcinoma as these markers have a varied sensitivity of 60-75% in primary breast carcinomas [9,10].

HER2/neu expression can be seen in both carcinomas of breast and lung [11]. Our findings with C-erb-2 protein score 2+/3+ and SISH without gene amplification have no differential value in this case. We found weak postive reaction for PGR in the core biopsies. Positive reactions for both ER and PGR have been reported for lung cancer [12] and give therefore no support to either breast cancer or metastasis diagnosis.

In agreement with Ersahin et al we found a positive expression of the basal-like marker CK5/6. This was especially noticeable in the in situ component, but only scattered positive cells in the invasive component. This can possibly represent a dedifferentiation in the tumour tissue. Previous studies have considered ductal carcinoma in situ with basal- like phenotype as a possible precursor to invasive basal-like cancer [13,14].

Our case showed high grade ductal carcinoma in situ and invasive tumour, and such tumours are more commonly basal like than low to intermediate grade lesions [15].

## Conclusion

We have presented an unusual case of TTF-1 positive primary infiltrating ductal carcinoma with focal basal-like and neuroendocrine differentiation. The in situ component with positive TTF-1 is the most important indication that this represents a primary tumour. Despite the fact that TTF-1 is known as a specific marker for lung and thyroid tumours, this case shows that primary non small-cell breast carcinomas can also be positive.

## Competing interests

The authors declare that they have no competing interests.

## Authors' contributions

TK wrote the manuscript and participated in histological diagnosis. YC participated in histological diagnosis and writing. MG supplied the relevant radiological details and participated in writing. HA operated the patient and supplied the relevant clinical details. BW participated in immunohistochemical analysis. TS participated in histological diagnosis and reviewed the manuscript. All authors have read and approved the final manuscript.

## Consent

Written informed consent was obtained from the patient for publication of this case report and accompanying images. A copy of the written consent is available for review by the Editor-in-Chief of this journal.
